# Supplementary material for: Future changes in severe hail across Europe, including regional emergence of warm-type thunderstorms
Source: Nat Commun. 2025 Sep 26;16:8438. doi: 10.1038/s41467-025-62780-0 (PMC12475258; doi:10.1038/s41467-025-62780-0)
Supplement: Supplementary file 1 — Supplementary Information [file 41467_2025_62780_MOESM1_ESM.pdf]

Supplementary Information

Future changes in severe hail across Europe including regional emergence of warm-type thunderstorms

Abdullah Kahraman[1,2,3,*], Elizabeth J. Kendon[4,5], Hayley J. Fowler[1,3], Chris J. Short[4]

[1]School of Engineering, Newcastle University, Newcastle upon Tyne, UK

[2]Visiting scientist at Met Office Hadley Centre, Exeter, UK

[3] Tyndall Centre for Climate Change Research, Newcastle University, Newcastle upon Tyne, UK

[4]Met Office Hadley Centre, Exeter, UK

[5] Bristol University, Bristol, UK

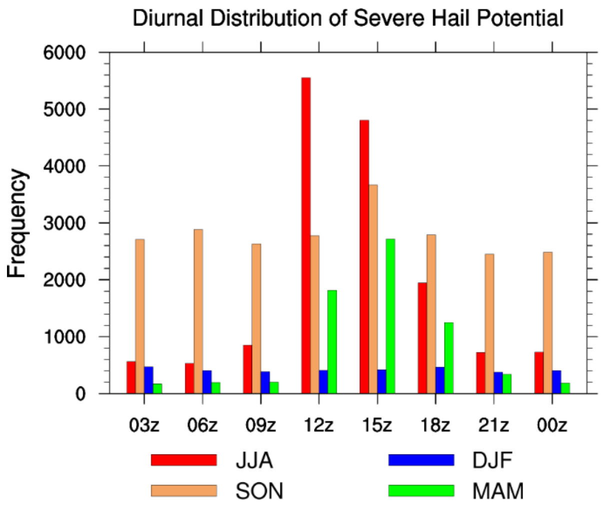

Supp. Figure 1: Diurnal distribution of severe hail potential frequency for December-January-February (DJF), March-April-May (MAM), June-July-August (JJA), and September-October-November (SON), for whole domain (excluding 70 grid points from lateral boundaries).

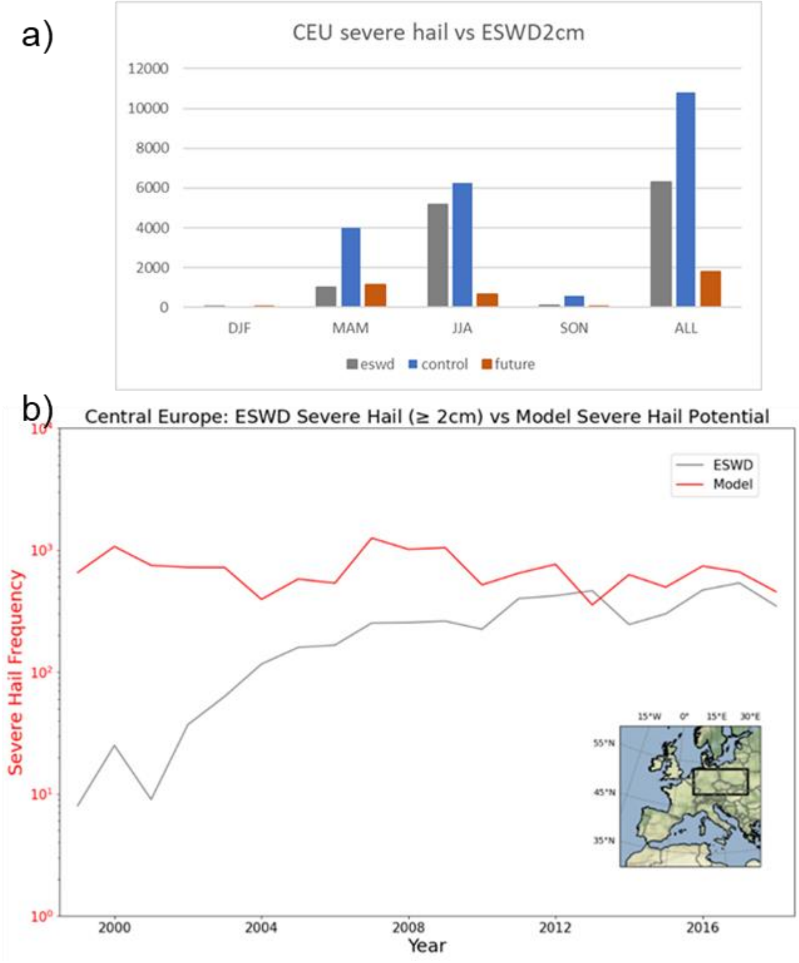

Supp. Figure 2: a) Number of ESWD reports with 2 cm or larger hail in the last 10 years (2012-2021), and severe hail potential in both 10-year simulations in Central Europe (CEU region shown in b), for December-January-February (DJF), March-April-May (MAM), June-July-August (JJA), September-October-November (SON), and all year (ALL). b) Number of ESWD severe hail reports in Central Europe (≥ 2 cm in diameter) and the frequency of severe hail potential extracted from the hindcast simulation for the same area from 1999 to 2018 (number of grid points satisfying the SHP criteria per year). The analysis area is depicted in the lower right panel as a black box in the domain map, subfigure adapted from Kahraman et al. 2024 study[29].

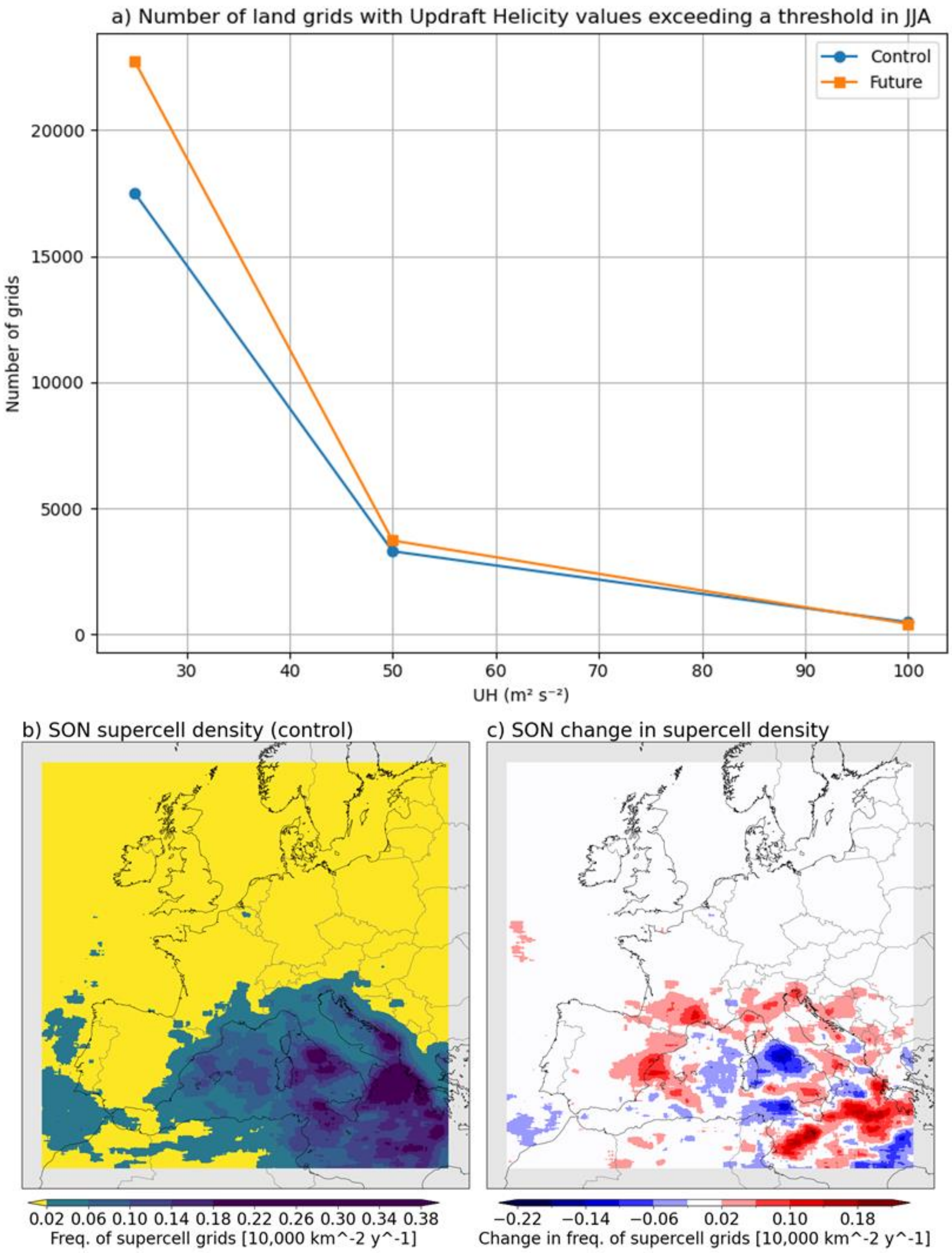

Supp. Figure 3: a) Number of land grids with Updraft Helicity (UH) values exceeding a threshold in JJA for both control and end-of-century future simulations. b) SON supercell density in the control simulation, based on an UH threshold of 50 m^2 s^{-2}. c) Future (end-of-century) changes in supercell density for SON. Supercells are defined as updrafts with strong rotation, i.e. updraft helicity values of 50 m^2 s^{-2} and above which is calculated using vertical velocity and relative vorticity within the 850 hPa-700 hPa and 700 hPa-500hPa layers, for 10 years of future simulation and 10 years of current simulation, using 3-hourly shapshots. Supercell density is defined as the number of supercells per 10,000 km^2 area. A smoothing has been applied by averaging the neighbouring ±25 grid points in b) and c). 70 grids from each lateral boundary are excluded from the analysis to remove boundary artefacts (shaded with grey).

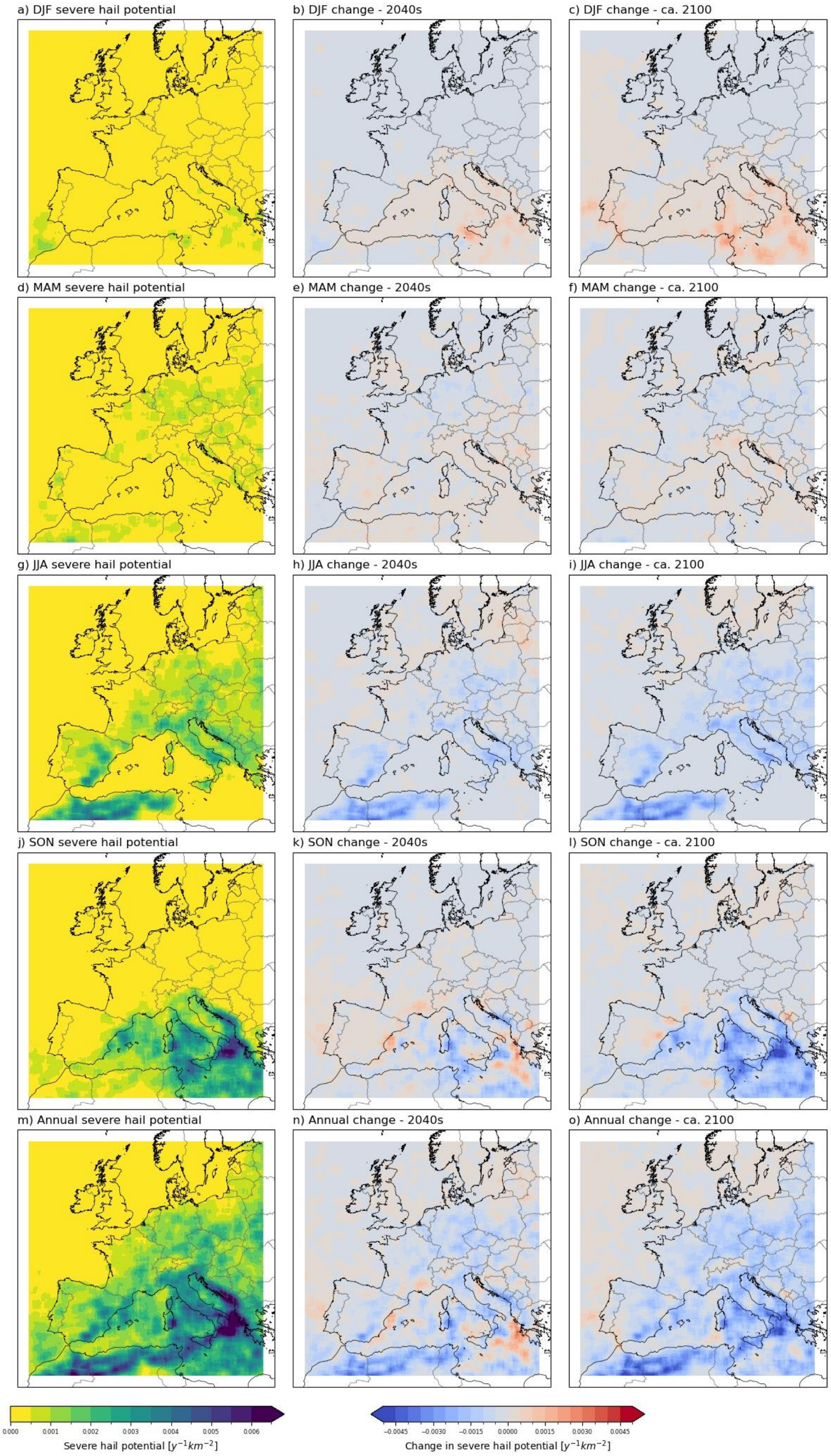

Supp. Figure 4: Severe hail potential in the current climate, and future (2040s and end-of-century) changes in severe hail potential, for DJF (a, b, and c, respectively), for MAM (d, e, f), for JJA (g, h, i), for SON (j, k, l) and for whole year (m, n, o). The current climate corresponds to 1998-2007, and future climates are comparable 10-year simulations corresponding to 2040-2049 and the year 2100 under RCP8.5. A smoothing has been applied by averaging the neighbouring ±25 grid points. 70 grids from each lateral boundary are excluded from the analysis to remove boundary artefacts.

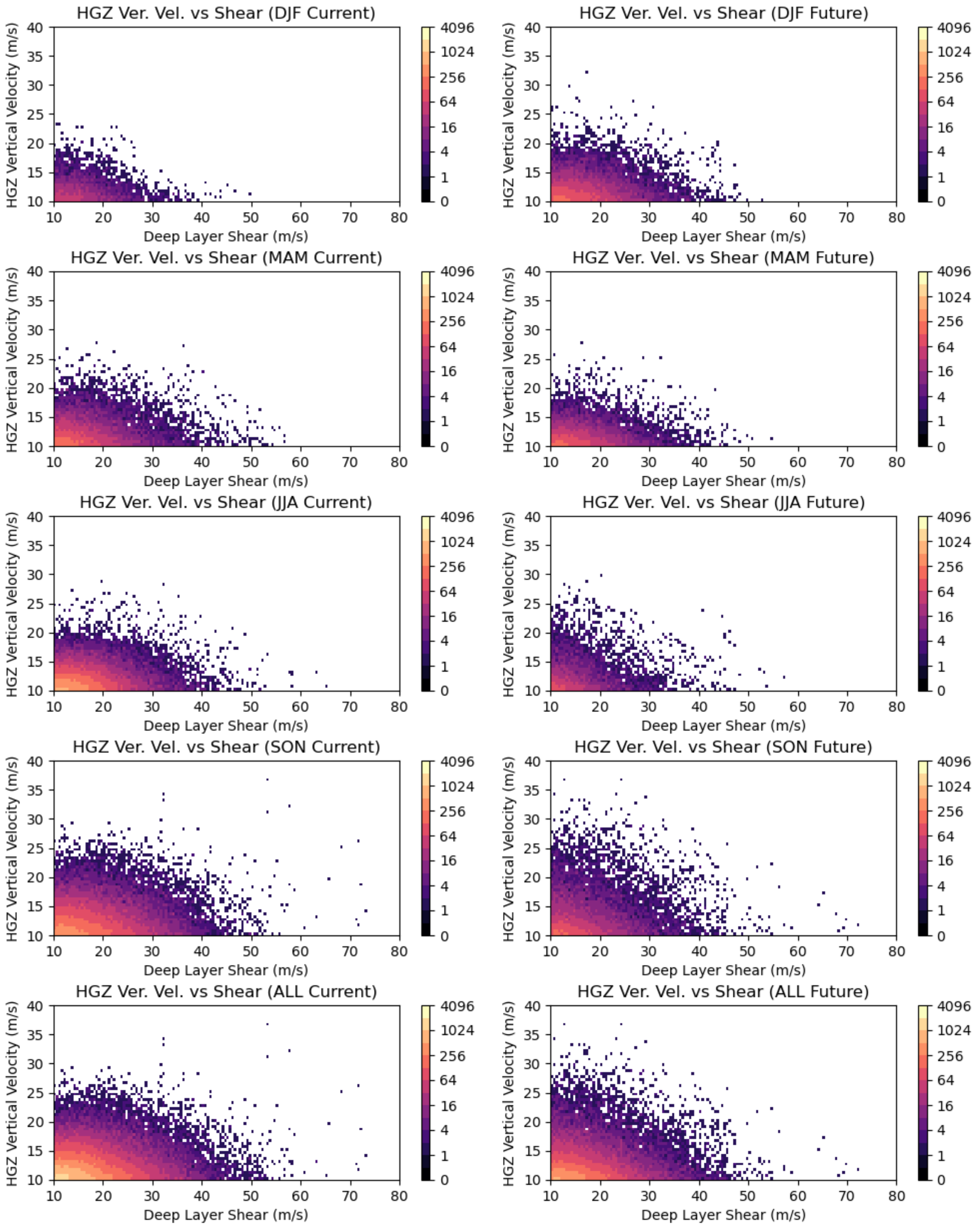

Supp. Figure 5: Seasonal and annual 2D histograms for vertical velocity in the hail growth zone vs deep layer shear during severe hail potential for current and future (end-of-century) simulations. A statistical significance test is not applied here to highlight rare, extreme values, which are important especially for significant severe hail potential.

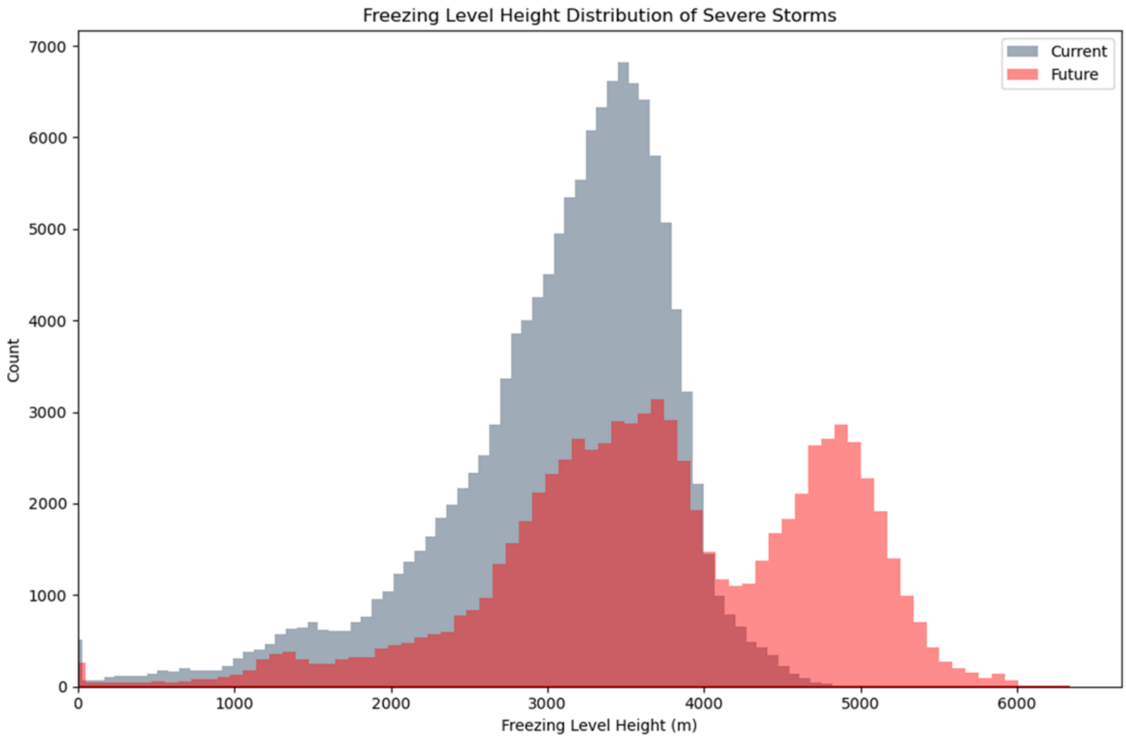

Supp. Figure 6: Freezing level height distribution of thunderstorms (grids exceeding graupel threshold) with high vertical velocity in the hail growth zone, with high vertical wind shear (as in Fig. 4 but for all year). The dark red area is the overlap of current (grey) and future (red) data.

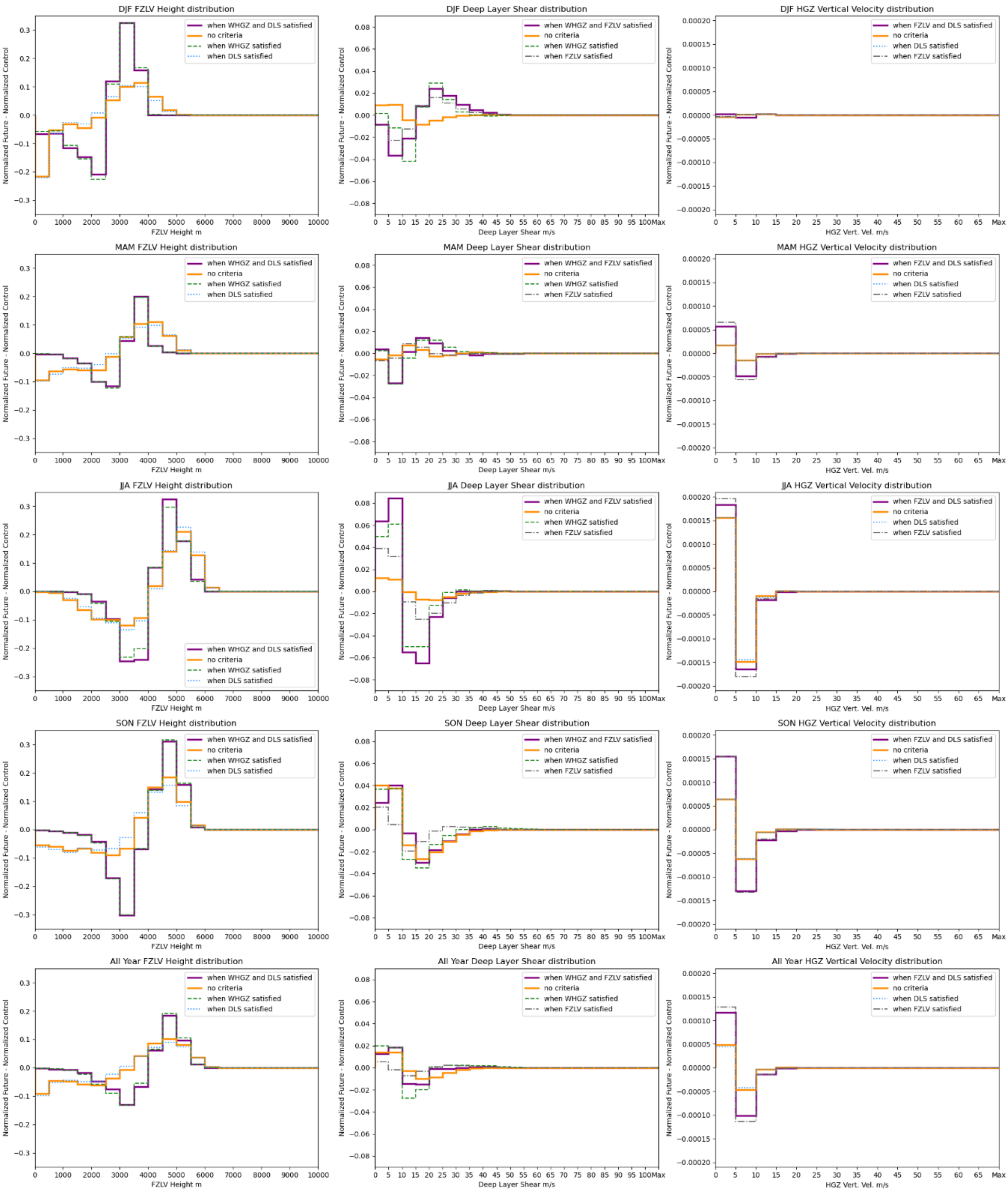

Supp. Fig. 7: As in Fig 7, but for all seasons and whole year, for all possible combinations: Future changes (end-of-century minus control) in normalised distributions of Severe Hail Proxy components, when applied separately, and when restricted by one or more other component's threshold range.

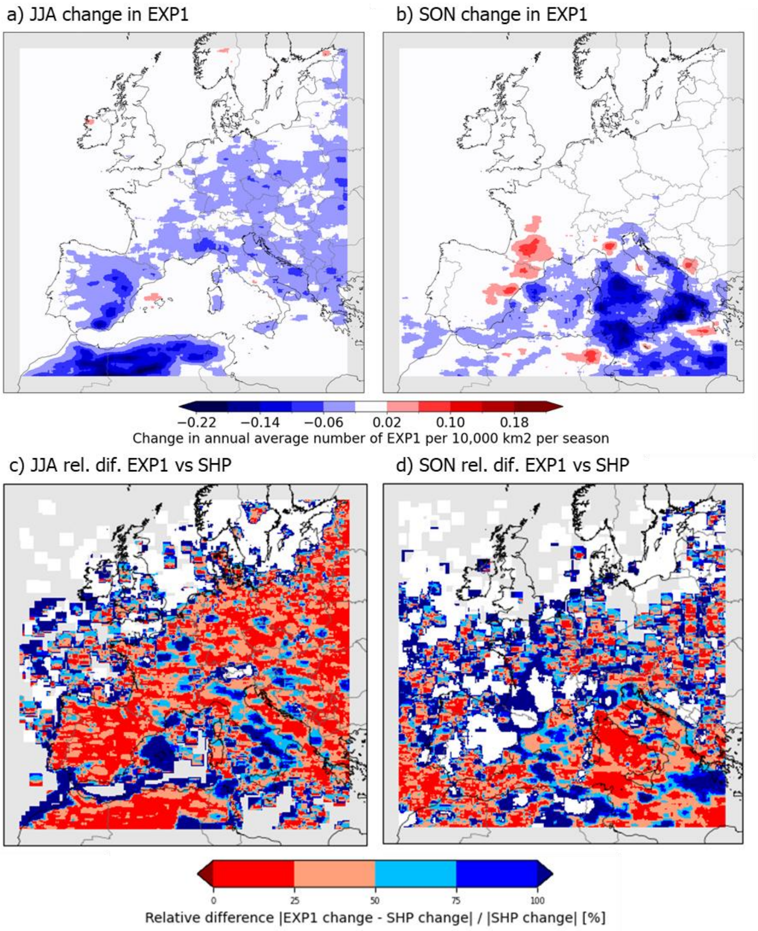

Supp. Figure 8: Future change in EXP1 (Severe Hail Potential without freezing level height criteria, i.e. with Graupel, HGZ vertical velocity and deep layer shear thresholds only) a) for JJA, b) for SON. Relative difference between future change in EXP1 and SHP ([EXP1 change – SHP change]/|SHP change|) c) for JJA and d) for SON.

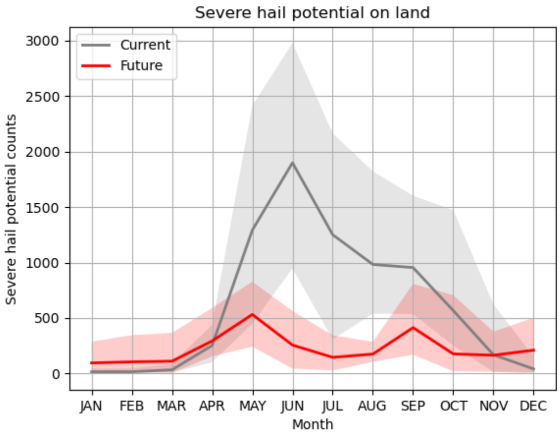

Supp. Figure 9: Severe hail potential per month in the current and future simulations on land grids. The grey and red shaded areas span the 10 years sampled within the current and future simulations respectively, and lines depict the average values. The analysis is excludes 70 grids from each lateral boundary.
